# Lowered Rhythm Tapping Ability in Patients With Constructional Apraxia After Stroke

**DOI:** 10.3389/fnins.2020.00247

**Published:** 2020-03-24

**Authors:** Naomi Kobinata, Hideto Yoshikawa, Yuji Iwasaka, Nobuyuki Kawate

**Affiliations:** ^1^Department of Rehabilitation Medicine, School of Medicine, Showa University, Tokyo, Japan; ^2^Department of Rehabilitation, Yoshieikai Hospital, Osaka, Japan; ^3^Department of Medicine, Tokyo Chidori Hospital, Tokyo, Japan; ^4^Department of Physical Therapy, Nihon Institute of Medical Science, Saitama, Japan

**Keywords:** rhythm tapping, constructional apraxia, synchronization, temporal reproduction, spatial reproduction

## Abstract

Rhythm tapping tasks are often used to explore temporal reproduction abilities. Many studies utilizing rhythm tapping tasks are conducted to evaluate temporal processing abilities with neurological impairments and neurodegenerative disorders. Among sensorimotor and cognitive disorders, rhythm processing abilities in constructional apraxia, a deficit in achieving visuospatial constructional activities, has not been evaluated. This study aimed to examine the rhythm tapping ability of patients with constructional apraxia after a stroke. Twenty-four patients were divided into two groups: with and without constructional apraxia. There were 11 participants in the constructional apraxia group and 13 in the without constructional apraxia group. The synchronization-continuation paradigm was employed in which a person performs a synchronized tapping activity to a metronome beat and continues tapping after the beat has stopped. For statistical analysis, a three-way mixed analysis of variance (2 × 2 × 3) was conducted. The factors were groups (with and without constructional apraxia), tapping tasks (synchronization and continuation), and inter-stimulus intervals (600, 750, and 1000 ms). A significant effect of group factor was found (*F*[1,132] = 16.62; *p* < 0.001). Patients in the without constructional apraxia group were able to more accurately reproduce intervals than those in the constructional apraxia group. Moreover, a significant effect of tapping tasks was found (*F*[1,132] = 8.22; *p* < 0.01). Intervals were reproduced more accurately for synchronization tasks than continuation tasks. There was no significant inter-stimulus interval effect. Overall, these results suggest that there might be a relation between temporal and spatial reproductions in a wide spectrum of processing levels, from sensory perception to cognitive function.

## Introduction

Rhythm tapping tasks are often used to explore temporal reproduction abilities (see Repp and Su, 2013, for a review). It is often performed as a finger tapping task in synchrony with an external rhythm, usually a steady metronome beat (Repp and Su, 2013). Along with the synchronization paradigm, synchronization-continuation tasks are often used to assess entrainment to an external rhythm (Flach, 2005; Ullén et al., 2008; Avanzino et al., 2013; McPherson et al., 2018). With synchronization-continuation tasks, individuals tap in synchrony to an external beat and continue tapping after the external beat has stopped (Flach, 2005).

In synchronization tasks, automatic or cognitive control is involved depending on the speed of the external beat (Miyake et al., 2004; Repp and Su, 2013; Bååth et al., 2016). In time perception research, sub-second time processing is automatic and supra-second time processing involves cognitive control (Bååth et al., 2016). For example, Mangels et al. (1998) showed that patients with prefrontal lesions who had difficulty with a non-temporal working memory task also struggled with long duration temporal discrimination (4-s interval) but not with short duration temporal discrimination (400-ms interval). Miyake et al. (2004) conducted a study employing the dual tasks of synchronization tapping and word-memory; they found that with anticipatory tapping, synchronization with a stimulus interval of 1800 to 3600 ms was affected by a word-memory task but not synchronization with a stimulus interval of 1500 ms or less. Similar results were found with dual tasks involving executive control (Bååth et al., 2016).

Neural mechanisms for time measurement support the available behavioral evidence. Measurements of sub-second intervals revealed activity in the bilateral supplementary motor area, left sensorimotor cortex, right cerebellum, right lateral premotor area, left thalamus, left basal ganglia, and right superior temporal gyrus (Lewis and Miall, 2003). In cognitively controlled timing tasks, the right prefrontal and parietal cortices were involved in addition to some parts of the autonomic system (right premotor area and bilateral supplementary motor area) (Lewis and Miall, 2003).

Compared to the synchronization paradigm, synchronization-continuation requires internal pacing without external cues and increases the neural resources required (Serrien, 2008). In Serrien (2008)’s study, electroencephalogram coherence increased in mesial-central connections under the continuation condition. Moreover, Ullén et al. (2008) reported a correlation between tapping stability and the volume of the right prefrontal white matter regions under a continuation condition. These studies show that performing a continuation task requires internal control and increases neural activities. Unlike the synchronization task, the continuous sub-second tapping task requires cognitive control. According to Ullén et al. (2008), intelligence and the stability of continuous sub-second tapping were correlated; also, Holm et al. (2017) reported that executive control and working memory were involved in continuous sub-second tapping.

Many studies utilizing rhythm tapping tasks are conducted to evaluate temporal processing abilities with neurological impairments and neurodegenerative disorders (Freeman et al., 1993; Schwartze et al., 2011, 2016; Avanzino et al., 2013; Roalf et al., 2018). Schwartze et al. (2016) reported that patients with cerebellar lesions display imprecise temporal processing compared to healthy participants in a control group. Similar results were reported with patients with basal ganglia lesions that might have impaired attention-dependent temporal processing (Schwartze et al., 2011). Furthermore, with Parkinson’s disease, temporal processing impairments were discussed in association with abnormalities of internal rhythm generation (Freeman et al., 1993) and motor planning impairments (Avanzino et al., 2013). Besides these reports, studies have shown time processing impairments in cases of Huntington’s disease (Agostino et al., 2017), Alzheimer’s disease (Roalf et al., 2018), mild cognitive impairment (Roalf et al., 2018), attention deficit hyperactivity disorder (Hove et al., 2017), and aphasia (Zipse et al., 2014).

Among sensorimotor and cognitive disorders, rhythm processing abilities in constructional apraxia have not been examined. Constructional apraxia is defined as a deficit in performing visuospatial constructional activities (Cubelli and Della Sala, 2018; Gainotti and Trojano, 2018) such as 2- or 3-dimensional copying or reproducing a drawing from memory and re-arranging patterns by blocks or sticks (Laeng, 2006; Russell et al., 2010); it is caused by cerebrovascular diseases such as stroke or brain damage on either hemisphere or neurodegenerative diseases such as Alzheimer’s disease (Mack and Levine, 1981; Trojano et al., 2004; Laeng, 2006; Gainotti and Trojano, 2018). With stroke patients, lesion sites associated with constructional apraxia include the basal ganglia, thalamus, posterior parietal lobule, lingual gyrus, calcarine, insula, temporal gyrus, temporo-parietal junction (Chechlacz et al., 2014), parietal lobes, frontal lobes, and occipital lobes (Cubelli and Della Sala, 2018). Notably, various regions of the brain are involved in the drawing process. Therefore, constructional apraxia is related to a broad range of symptoms including: dysfunctions in visuospatial abilities such as the processing of shapes and the interrelations between different components of objects, perception, attentional allocation to global and local features, executive functions such as planning, and motor mechanisms (Chechlacz et al., 2014; Gainotti and Trojano, 2018).

Based on studies on lowered cognitive abilities with constructional apraxia (Laeng, 2006; Chechlacz et al., 2014; Nagaratnam et al., 2014; Gainotti and Trojano, 2018) and on the involvement of cognitive control such as general intelligence, working memory, and executive control on temporal reproduction (Ullén et al., 2008; Holm et al., 2017), it is likely that patients with constructional apraxia would show lowered temporal processing that requires cognitive control. It is worth examining the automatic temporal processing abilities of patients with constructional apraxia, including impairments in visuospatial perception, given the shared temporal and spatial performance and shared neural resources in sensorimotor synchronization (Doumas and Wing, 2007; Comstock et al., 2018), the common magnitude system in spatial lines and temporal duration representation (De Corte et al., 2017), the left-to-right ordering system (Bonato et al., 2016), and the temporal coding of visual spaces (Rucci et al., 2018).

The current study aims to examine the rhythm tapping ability of patients with constructional apraxia after a stroke. The performance of patients was examined during synchronization and continuation tapping tasks with sub-second stimulus intervals. If the patients demonstrated a lowered ability to synchronize with sub-second stimulus intervals, then their automatic timing process was regarded as lowered. If the patients’ sub-second continuation tapping was less accurate than those without constructional apraxia, then a deficit in cognitive control on temporal reproduction was suggested. Based on previous studies (Laeng, 2006; Doumas and Wing, 2007; Ullén et al., 2008; Chechlacz et al., 2014; Nagaratnam et al., 2014; Bonato et al., 2016; De Corte et al., 2017; Holm et al., 2017; Comstock et al., 2018; Gainotti and Trojano, 2018; Rucci et al., 2018), we hypothesized that patients with constructional apraxia would perform less accurately with both sub-second synchronization and continuation tapping tasks than those without constructional apraxia.

## Materials and Methods

In this retrospective study, clinical records of stroke patients admitted to a post-acute rehabilitation unit in Japan between November 2012 and February 2015 were queried for results of constructional apraxia tests and finger tapping tasks. The finger tapping tasks performed during this period were conducted to examine the ability of patients to synchronize to auditory stimulation. This study was approved by the ethical committee of Shimousa Hospital and conducted in accordance with the Declaration of Helsinki. The requirement of informed consent was waived. Instead, the patients were provided with the opportunity to opt out after posting the purpose and method of this research.

### Patients

There were 44 eligible patients who performed constructional apraxia tests and finger tapping tasks. Data were excluded from 20 patients according to the following exclusion criteria: a prior stroke episode, bilateral lesions, a strong influence of unilateral neglect on drawing, a disturbance of consciousness, and a failure to complete the assessments. Of the 44 patients, data were analyzed from 24 patients. These 24 patients were divided into two groups: with or without constructional apraxia. Eleven patients were allotted to the constructional apraxia group and 13 were assigned to the without constructional apraxia group. Characteristics of the patients are described in Tables 1, 2. Lesion sites were diverse in both groups. Regarding the lesioned brain hemispheres, nine patients had damage on the right side and two had damage on the left side in the constructional apraxia group. In the without constructional apraxia group, five had damage on the right side and eight had damage on the left side. The proportion of affected dominant hands was determined by the proportion of lesioned right and left hemispheres. In the constructional apraxia group, two had affected dominant hands and nine had unaffected dominant hands. In the without constructional apraxia group, eight had affected dominant hands and five had unaffected dominant hands. The mean motor and cognition subscale values of the Functional Independence Measure (FIM) for the constructional apraxia group were 39.2 and 14.2, respectively, and the values for the without constructional apraxia group were 55.5 and 23, respectively. The FIM consists of 18 items and is grouped into motor and cognition subscales. The value of the total score for the motor subscale is between 13 and 91 and that for the cognition subscale is between 5 and 35 (Hamilton et al., 1994).

**TABLE 1:** Description of the participants in the constructional apraxia group.

Patient	Age range	Post-stroke day	Lesioned hemisphere	Dominant hand	Affected side	FIM motor score	FIM cognition score	Diagnosis
1	66–70	53	Right	Right	Left	29	20	Corona radiata infarction
2	71–75	59	Right	Right	Left	33	11	Fronto-temporal lobe infarction
3	66–70	209	Right	Right	Left	51	16	Internal capsule and corona radiata infarction
4	76–80	146	Right	Right	Left	81	22	Occipital lobe, thalamic infarction
5	21–25	30	Right	Right	Left	25	12	Putaminal hemorrhage
6	61–65	36	Right	Right	Left	20	13	Internal carotid artery territory infarction
7	71–75	73	Right	Right	Left	60	15	Occipital lobe and thalamic infarction
8	71–75	35	Right	Right	Left	32	13	Temporal lobe and corona radiata infarction
9	81–85	58	Left	Right	Right	24	11	Frontal lobe hemorrhage
10	75–80	23	Right	Right	Left	35	17	Thalamic hemorrhage
11	75–80	54	Left	Right	Right	41	6	Frontal subcortical infarction
Mean	69.8	70.6				39.2	14.2	
Standard deviation	15.9	56.6				18.3	4.5	

*FIM motor, Functional Independence Measure motor subscale (a value between 13 and 91); FIM cognition, Functional Independence Measure cognition subscale (a value between 5 and 35).*

**TABLE 2:** Description of the participants in the without construction apraxia group.

Patient	Age range	Post-stroke day	Lesioned hemisphere	Dominant hand	Affected side	FIM motor score	FIM cognition score	Diagnosis
1	66–70	173	Right	Right	Left	52	30	Parietal lobe infarction
2	71–75	81	Right	Right	Left	25	25	Pontine and medullary infarction
3	76–80	31	Left	Right	Right	83	31	Parietal lobe infarction
4	66–70	60	Left	Right	Right	31	11	Corona radiata, parietal lobe and cerebellar infarction
5	51–55	40	Left	Right	Right	59	14	Subarachnoid hemorrhage, parietal-occipital lobe, and thalamic infarction, corpus callosum infarction
6	71–75	84	Right	Right	Left	80	27	Frontal subcortical and occipital lobe infarction
7	66–70	69	Right	Right	Left	78	25	Frontal subcortical infarction
8	41–45	81	Left	Right	Right	88	28	Frontal lobe hemorrhage
9	46–50	23	Right	Right	Left	53	25	Brainstem and cerebellar infarction
10	81–85	25	Left	Right	Right	33	21	Watershed infarction
11	61–65	20	Left	Right	Right	46	23	Corona radiata infarction
12	56–60	45	Left	Right	Right	53	17	Thalamic hemorrhage
13	71–75	22	Left	Right	Right	40	22	Putaminal hemorrhage
Mean	65.5	58				55.5	23	
Standard deviation	11.6	42.2				21.1	6	

### Constructional Apraxia Test

**FIGURE 1:**
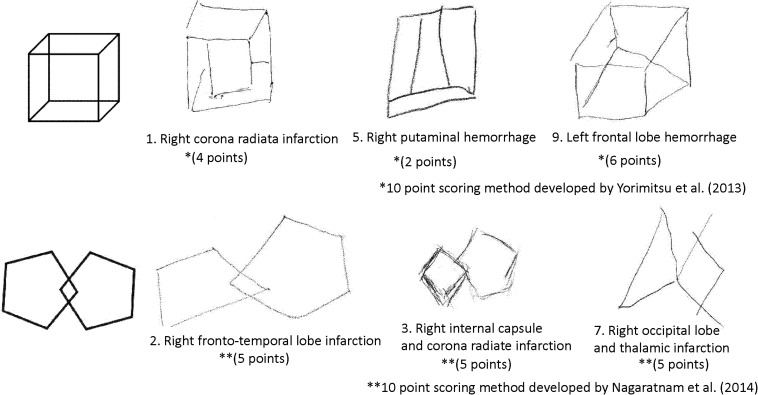
Sample drawings of a cube and an intersecting pentagon from the constructional apraxia group.

To determine the presence of constructional apraxia, the results of a cube copying test or an intersecting pentagon copying test were used except for a patient who did not have either test result but had performed well on the Rey-Osterrieth complex figure test. This patient was included in the without-constructional apraxia group. To assess the cube copying test results, the scoring method developed by Yorimitsu et al. (2013) was employed that involves a checklist of inadequacies such as a lack of line or depth and a distortion in shape or proportion. All individuals who scored 6 or less out of a 10-point scale were included in the constructional apraxia group. To evaluate the intersecting pentagon copying test results, the scoring method developed by Nagaratnam et al. (2014) was utilized; this is a 10-point scoring method based on the degree of drawing failure. Participants with an intersecting pentagon copying score of 8 or less were included in the constructional apraxia group. Sample drawings from the constructional apraxia group are presented in Figure 1.

### Finger Rhythm Tapping

**FIGURE 2:**
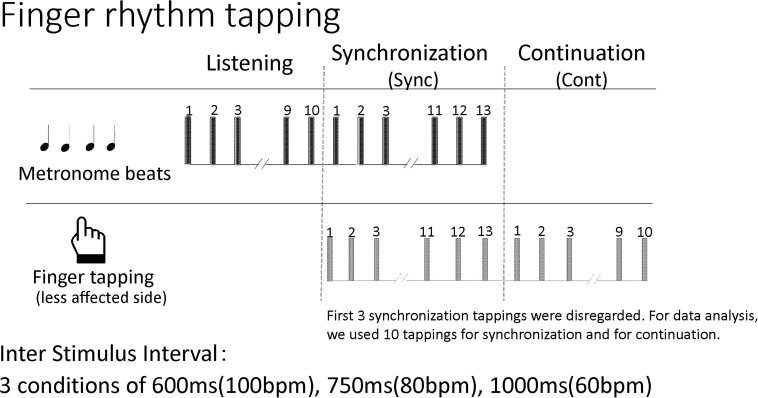
The procedure for the finger rhythm tapping task. bpm, beats per minute.

Participants completed a finger tapping task at least once during their hospitalization. The first dataset of individuals who completed the task twice was used. For this study, the synchronization-continuation paradigm was employed in which tapping is synchronized to a metronome beat and the patient continues tapping after the beat has stopped. The metronome sound of a Yamaha electronic keyboard EZ-J210, presented through a speaker, was used. The volume was set at a comfortable level for each participant. At first, patients listened to about 10 metronome beats and tapped in synchrony with the beats about 13 times. After the beat stopped, patients continued to tap at the same interval about 10 times. Patients repeated this procedure for three inter-stimulus intervals: 600 ms (100 metronome beats/min), 750 ms (80 metronome beats/min), and 1000 ms (60 metronome beats/min). There was no practice of the task prior to the assessment. Each condition was measured once without any repetition. In the finger tapping task, inter-stimulus intervals longer than 1000 ms are often used to examine cognitive involvement (Mangels et al., 1998; Miyake et al., 2004; Bååth et al., 2016). In this study, inter-stimulus intervals less than 1000 ms were chosen and the results of synchronization tasks were compared with those of continuation tasks. Figure 2 shows the procedure.

### Data Acquisition

Recorded metronome beats were used for each condition. The participants wore a plastic finger pick on the index finger of their less affected side and tapped on a hard surface next to the touchpad on a laptop computer. The metronome beats and taps were recorded using audio editing software Sound it! 6 (Internet Co., Ltd., Osaka, Japan).

### Data Analysis

Tap onset and metronome beats were identified with a waveform display using the software. The first 3 synchronization taps were disregarded and 10 taps were used for both the synchronization and continuation phases to calculate the interval reproduction accuracy index for each condition. The interval reproduction accuracy index is the ratio between the finger tapping interval reproduced by the person and the inter-stimulus interval set by the metronome. The method in the Avanzino et al. (2013) study served as a guide for the interval reproduction accuracy index (= the finger tapping interval reproduced by the person/the inter-stimulus interval set by the metronome). Briefly, when the tapping interval and inter-stimulus interval set by the metronome are equal, the interval reproduction accuracy index value equals 1. However, when the tapping interval is longer than the inter-stimulus interval, the index value is more than 1, and when it is shorter, the index value is less than 1.

### Statistics

Statistical analyses were performed with EZR (Saitama Medical Center, Jichi Medical University, version 1.35), a graphical user interface for R (The R Foundation for Statistical Computing, version 3.3.2) (Kanda, 2013). A 3-way mixed analysis of variance (ANOVA) (2 × 2 × 3) was performed for the interval reproduction accuracy index. The factors were groups (with and without constructional apraxia), tapping tasks (synchronization and continuation), and inter-stimulus intervals (600, 750, and 1000 ms). To evaluate how well the continuation performance was maintained from the synchronization condition, a 2-way mixed ANOVA (2 × 3) was performed for the interval reproduction index (the finger tapping interval reproduced by a participant for the continuation condition relative to the interval reproduced for the synchronization condition). When the tapping interval for the continuation and synchronization conditions are equal, the index value is 1. The factors were groups (with and without constructional apraxia) and inter-stimulus intervals (600, 750, and 1000 ms). Also, a *post hoc* statistical power analysis was conducted using the G^∗^Power 3.1.9.4 software (Christian-Albrechts-Universität Kiel, Kiel, Germany) (Faul et al., 2007) with the effect size (*f*) = 0.25, the significance level (α) = 0.05, and the power (1−β) = 0.8 for between-factor comparisons. For demographic differences between groups, both age and days from the onset were compared using a Welch’s *t*-test and the proportion of lesioned hemispheres and dominant hands were examined using a Fisher’s exact test. In addition, a 1-way analysis of covariance (ANCOVA) was conducted to determine statistically significant differences in the interval reproduction accuracy indexes controlling for lesioned hemispheres between groups (with and without constructional apraxia). Scores for FIM motor and cognition subscales were compared between groups using a Welch’s *t*-test. *P* values < 0.05 were considered statistically significant.

## Results

**FIGURE 3:**
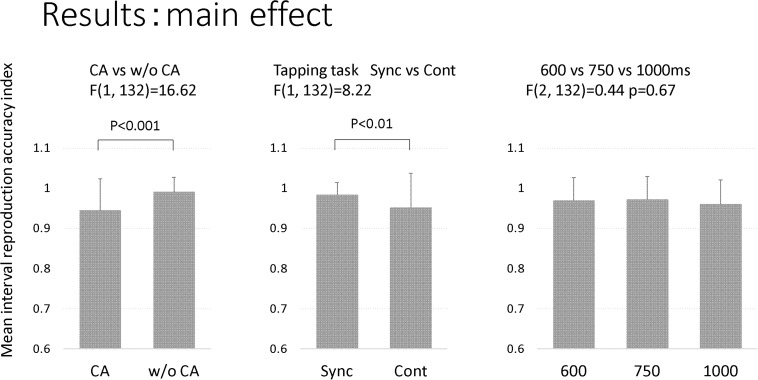
The main effect of the factors. The factors included groups (CA, constructional apraxia; w/o CA, without constructional apraxia), tapping tasks (Sync, synchronization; Cont, continuation), and inter-stimulus intervals (600 ms, 750 ms, and 100 ms). Error bars indicate standard deviations.

**FIGURE 4:**
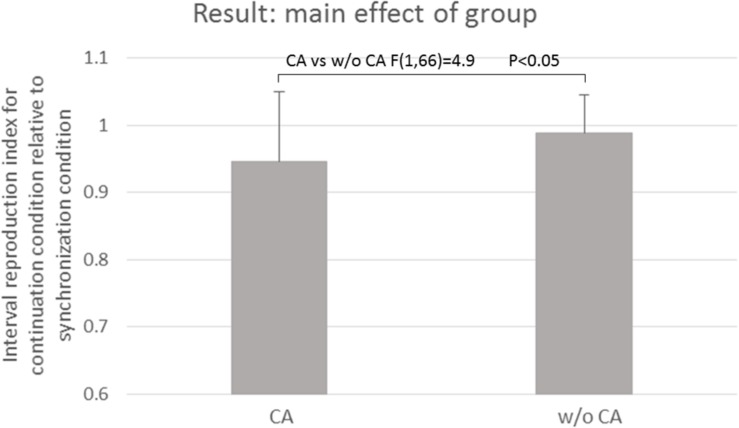
The main effect of the group. CA, constructional apraxia; w/o CA, without constructional apraxia. Error bars indicate standard deviations.

Table 3 and Figure 3 show the results of the mean reproduction accuracy index; Table 3 shows the mean and standard deviation for each condition while Figure 3 displays the main effects. There was a significant effect of group (*F*[1,132] = 16.62; *p* < 0.001); the without constructional apraxia group was able to more accurately reproduce the intervals than the constructional apraxia group. As expected, tapping tasks had a significant effect (*F*[1,132] = 8.22; *p* < 0.01); intervals were reproduced more accurately for synchronization tasks than continuation tasks. There was no significant effect of inter-stimulus intervals. A 2-way ANOVA revealed a significant main effect of group (*F*[1,66] = 4.9; *p* < 0.05); the without constructional apraxia group was able to more accurately reproduce the interval for the continuation condition relative to the interval reproduced for the synchronization condition. In other words, the without constructional apraxia group was able to maintain the continuation performance from the synchronization condition. The results of the ANOVA analysis are shown in Figure 4. There was no significant main effect of inter-stimulus intervals or interaction effect. The *post hoc* statistical power analysis revealed a value of 0.30 with a sample size of 24 for this study. This power analysis also revealed that a sample size of 86 would be needed to detect a medium size effect (f = 0.25; cf. Cohen, 1977) with 0.80 power (1−β) at the 0.05 statistical significance level.

**TABLE 3:** The mean and standard deviation of the interval reproduction accuracy index.

	Sync			Cont		
Interstimulus interval	600 ms	750 ms	1000 ms	600 ms	750 ms	1000 ms
CA	0.952 ± 0.063	0.982 ± 0.028	0.979 ± 0.041	0.947 ± 0.096	0.925 ± 0.132	0.881 ± 0.118
w/o CA	0.993 ± 0.020	0.997 ± 0.017	0.998 ± 0.015	0.988 ± 0.048	0.984 ± 0.054	0.982 ± 0.069

*CA, constructional apraxia; w/o CA, without constructional apraxia; Sync, synchronization; Cont, continuation.*

Concerning the demographic characteristics, there were no significant differences in the age or day from onset between the participants. There was a significant between-group difference in the proportion of the lesioned hemispheres. The proportion of right-side brain damage was higher in the constructional apraxia group than in the without constructional apraxia group (*p* < 0.05). Additionally, there was a significant between-group difference in the proportion of the affected dominant hands. The proportion of affected dominant hands was higher in the without constructional apraxia group than in the constructional apraxia group (*p* < 0.05). After controlling for lesioned hemispheres, the difference in interval reproduction accuracy between the groups was significant (*F*[1,141] = 12.61; *p* < 0.001). Also, there was a significant between-group difference in the FIM cognition subscale score (*p* < 0.001). However, there was no significant between-group difference in the FIM motor subscale score.

## Discussion

Patients in the constructional apraxia group were less able to accurately reproduce sub-second intervals than those in the without constructional apraxia group. This result indicates the lowered automatic timing process of patients with constructional apraxia. Regarding cognitive involvement in temporal processing, our results support those of previous studies (Serrien, 2008; Ullén et al., 2008; Holm et al., 2017). The FIM cognition subscale score of the constructional apraxia group was significantly lower than the scores observed in the without constructional apraxia group. In the continuation condition, the constructional apraxia group displayed less accurate temporal reproduction compared to the without constructional apraxia group. These results raise the possibility that spatial and temporal reproduction abilities are related to a wide range of processing levels.

However, it is also possible that a distinctive line between automatic and cognitive processes does not exist, and that cognitive control is also involved in sub-second synchronization tapping. Bååth et al. (2016) stated that synchronization to sub-second intervals requires executive control, although its involvement is less than that observed with synchronization to longer intervals. Notably, the discrete neural resources involved in automatic and cognitive processes are unclear. Constructional apraxia is related to cognitive activities such as working memory, executive control, and general intelligence, as well as spatial perception. In this study, the constructional apraxia group had a significantly lower FIM cognition subscale score than those in the without constructional apraxia group. The involvement of cognitive control might be sufficient to explain why the patients in the constructional apraxia group were less able to accurately reproduce temporal intervals in both the synchronization and continuation tasks.

Another finding in this study is related to the effect of sub-second inter-stimulus intervals. Several intervals were employed (600, 750, and 1000 ms) for the synchronization and continuation paradigms, but there was no significant effect of the inter-stimulus interval. This null finding agrees with previous rhythm tapping studies (see Repp, 2005; Repp and Su, 2013, for a review) and suggests that there are common mechanisms across populations in terms of sub-second inter-stimulus interval differences.

A limitation of this study is the limited statistical power due to the small sample size (*n* = 24). The power analysis revealed that a sample size of 86 would be needed to detect a medium-sized effect with the recommended statistical power. Therefore, it is important to be cautious when interpreting the present results and further study with an increased sample size is required.

## Conclusion

This study shows that patients with constructional apraxia display a lowered ability to synchronize and reproduce temporal intervals. Given the lowered temporal and spatial reproduction abilities in patients with constructional apraxia, there might be a relationship between temporal and spatial reproductions in a wide spectrum of processing levels including those for sensory-perception and cognition.

## Data Availability Statement

The raw data supporting the conclusions of this article will be made available by the authors, without undue reservation, to any qualified researcher.

## Ethics Statement

The studies involving human participants were reviewed and approved by the ethical committee of Shimousa Hospital. Written informed consent for participation was not required for this study in accordance with the national legislation and the institutional requirements.

## Author Contributions

NKo collected data, performed the statistics, and wrote the manuscript. HY, YI, and NKa contributed with critical feedback to shape the research and assisted with the writing of the manuscript through extensive editing.

## Conflict of Interest

The authors declare that the research was conducted in the absence of any commercial or financial relationships that could be construed as a potential conflict of interest.
